# Deciphering how ApoE shapes neurodevelopment

**DOI:** 10.1002/alz.090731

**Published:** 2025-01-03

**Authors:** Reeteka Sud

**Affiliations:** ^1^ ADBS Lab, National Institute of Mental Health and Neurosciences (NIMHANS), Bengaluru, Karnataka India

## Abstract

**Background:**

What does neurodevelopment look like when neurodegeneration is the outcome – this is overarching theme of investigations currently ongoing in our lab. The E4 isoform of ApoE protein is the most consistently replicated risk factor in Alzheimer’s Disease (AD). And yet, much remains unknown about the biological pathways that connect *APOE4* genotype with the development of pathology that eventually leads to AD, nor do we know how early in life these cellular alterations begin.

**Method:**

We derived neural precursor cells (NPCs) from induced pluripotent stem cells (IPSCs) that were CRISPR‐edited at the *APOE* locus. As these cells give rise to neurons and glia in the developing fetal brain, elucidating how ApoE affects these cells can highlight its possible effects on neurodevelopment. Proteomic analysis was performed to characterize the protein expression landscape in the NPCs subsequent to targeted deletion of E4 from a parent IPSC line of *APOE3/4* genotype. Differentially expressed proteins (DEPs) following mass spectrometric analysis were determined from the protein abundance fold change values obtained for each protein. Proteins which showed >1.5‐fold difference with FDR adjusted P‐value < 0.05 were considered differentially expressed.

**Result:**

CRISPR‐editing of E4 from the parent line revealed 98 differential expressed proteins. Of these, 54 were upregulated, and 44 were downregulated. Further analysis of the DEPs via STRING database showed that these changes primarily affect pathways linked to RNA processing, plasma membrane repair, and cytoskeleton organization.

**Conclusion:**

It is crucial to understand the developmental roles of ApoE and the isoform‐specific differences in its functions to better understand its function and dysfunction. Indeed, we find the effects of E4 extend beyond proteins considered central to AD pathology. Our results add to the growing body of literature on altered course of neurodevelopment from genetic risk factors in AD.

